# Immortalized mesenchymal stem cells: an alternative to primary mesenchymal stem cells in neuronal differentiation and neuroregeneration associated studies

**DOI:** 10.1186/1423-0127-18-87

**Published:** 2011-11-25

**Authors:** Min Gong, Yang Bi, Wei Jiang, Yun Zhang, Li Chen, Nali Hou, Youxue Liu, Xiaoping Wei, Jie Chen, Tingyu Li

**Affiliations:** 1 Children's Nutrition Research Center, Children's Hospital of Chongqing Medical University, Chongqing 400014, China; 2 Ministry of Education Key Laboratory of Child Development and Disorders; Key Laboratory of Pediatrics in Chongqing; Chongqing International Science and Technology Cooperation Center for Child Development and Disorders, Chongqing 400014, China

**Keywords:** mesenchymal stem cells, reversible immortalization, simian virus 40 large T, cell senescence, neuronal differentiation, cell transplantation, hypoxic-ischemic brain damage

## Abstract

**Background:**

Mesenchymal stem cells (MSCs) can be induced to differentiate into neuronal cells under appropriate cellular conditions and transplanted in brain injury and neurodegenerative diseases animal models for neuroregeneration studies. In contrast to the embryonic stem cells (ESCs), MSCs are easily subject to aging and senescence because of their finite ability of self-renewal. MSCs senescence seriously affected theirs application prospects as a promising tool for cell-based regenerative medicine and tissue engineering. In the present study, we established a reversible immortalized mesenchymal stem cells (IMSCs) line by using SSR#69 retrovirus expressing simian virus 40 large T (SV40T) antigen as an alternative to primary MSCs.

**Methods:**

The retroviral vector SSR#69 expressing simian virus 40 large T (SV40T) antigen was used to construct IMSCs. IMSCs were identified by flow cytometry to detect cell surface makers. To investigate proliferation and differentiation potential of IMSCs, cell growth curve determination and mesodermal trilineage differentiation tests were performed. Neuronal differentiation characteristics of IMSCs were detected *in vitro*. Before IMSCs transplantation, we excluded its tumorigenicity in nude mice firstly. The Morris water maze tests and shuttle box tests were performed five weeks after HIBD models received cells transplantation therapy.

**Results:**

In this study, reversible IMSCs were constructed successfully and had the similar morphology and cell surface makers as primary MSCs. IMSCs possessed better ability of proliferation and anti-senescence compared with primary MSCs, while maintained multilineage differentiation capacity. Neural-like cells derived from IMSCs had similar expressions of neural-specific genes, protein expression patterns and resting membrane potential (RMP) compared with their counterparts derived from primary MSCs. There was no bump formation in nude mice subcutaneously injected with IMSCs. IMSCs played same role as primary MSCs to improve learning ability and spatial memory of HIBD rats.

**Conclusions:**

IMSCs not only retain their features of primary MSCs but also possess the ability of high proliferation and anti-senescence. IMSCs can definitely be induced to differentiate into neuronal cells *in vitro *and take the place of primary MSCs for cell transplantation therapy without tumorigenesis *in vivo*. The stable cell line is particularly useful and valuable as an alternative to MSCs in neuronal differentiation and neuroregeneration associated studies.

## Background

Mesenchymal stem cells (MSCs) are adult stem cells present in many tissues, such as bone marrow, adipose tissue and peripheral blood. They are able to differentiate into multiple mesodermal lineage cells, such as osteocytes, chondrocytes and adipocytes [1,2]. However, recent studies have demonstrated that MSCs have the ability to transdifferentiate across embryonic boundaries [3,4] and be induced to differentiate into nonmesodermal cells such as hepatocytes [5], endothelial cells [6] and neuronal cells [7,8] under appropriate environmental conditions both *in vitro *and *in vivo*. Our previous studies have clearly demonstrated that MSCs can be induced to differentiate into neuronal cells [9] and be used to improve neurological function in animal models of hypoxic-ischemic brain damage (HIBD) by cell transplantation therapy [10].

In contrast to the embryonic stem cells (ESCs) which derive from blastocyst early-stage embryos and possess an infinite capacity of self-renewal [11], MSCs have to be subject to aging and senescence as the gradual degradation of their replication ability [12]. Senescent MSCs have very typical morphological characteristics. They become flat, big and hypertrophic and reduce adhesion to plastic surfaces. Because of lipofuscin accumulation, autofluorescence level of senescent MSCs will increase. And then, they present constrained nuclei, granular cytoplasm and much cellular debris [13]. The proliferation ability and capacity to differentiate into various types of cells seems to decrease due to senescence. Although the underlying molecular mechanism of MSCs senescence is currently unclear, recent studies indicated that telomeres length [14], telomerase activity [15], extracellular signal-regulated kinases (ERK) signaling [16], TGF-β superfamily [17] and oxidative stress [18] were involved in MSCs senescence. MSCs senescence seriously affected its application prospects as a promising tool for cell-based regenerative medicine and tissue engineering. Therefore, it is a challenge in MSCs research to develop techniques for obtaining a sufficient number of MSCs out of limited passages and avoiding cell senescence.

In the present study, we infected primary MSCs with retrovirus that express simian virus 40 large T (SV40T) antigen to construct an immortalized mesenchymal stem cells (IMSCs) line. We found that the morphology and surface makers of high-passage IMSCs were the same as that of primary MSCs. The proliferation capacity of IMSCs is stronger than that of primary MSCs, meanwhile IMSCs maintained the mesodermal trilineage differentiation potentials. In the further neuronal differentiation research, we found that IMSCs had similar differentiation ability and maturation time pattern compared with primary MSCs and could be transplanted into brain injury animal models to improve its neurological function without tumor oncogenesis. In general, our data suggest that IMSCs might be a good and valuable alternative to MSCs in neural studies both *in vitro *and *in vivo*.

## Materials and methods

### Isolation and expansion of rat primary mesenchymal stem cells

Rat primary MSCs were isolated and expanded as described before [9]. In brief, after the sacrifice of 4-week-old Sprague Dawley (SD) rats, the tibias and femurs were removed and cleaned of connective and soft tissues. Bone marrow was flushed out of the cut bones after removal of the epiphysis. Cells were cultured in Dulbecco's modified Eagle medium/Ham's Nutrient Mixture F12 (DMEM/F12; Gibico) supplemented with 10% fetal bovine serum (FBS; Gibico), 100 units/ml penicillin, and 100 μg/ml streptomycin at 37°C in a humidified incubator containing 5% CO_2_. Cells were digested with trypsin (Invitrogen) and passaged at approximately 90% confluence every 3-4 days. Cells between passages three and six were used for subsequent experiments.

### Flow cytometry

Cultured cells were trypsinized and resuspended in PBS containing 1% bovine serum albumin (BSA). Cell suspensions were incubated with different antibodies including CD34-FITC (Santa Cruz), CD29-FITC (BD Biosciences), CD44-FITC (Santa Cruz), CD45-PE (Biolegend), CD90-PE (Biolegend), and CD106-PE (BD Biosciences) at recommended dilution for 30 min at room temperature in the dark. After washed with PBS twice, labeled cells were analyzed by FACSC-anto II system (BD Biosciences) and quantified by using CellQuest Pro software (BD Biosciences).

### Construction of reversible immortalized mesenchymal stem cells

The retroviral vector SSR#69 that expresses SV40T antigen and hygromycin resistant gene flanked with loxP sites [19] and package vector pAmpho were co-transfected into human embryonic kidney 293 (HEK293) cell lines by using LipfectMINE 2000 to package retrovirus expressing SV40T antigen. Freshly isolated MSCs were seeded in T25 flask and infected with 2 mL of filtered retrovirus containing 4 μg/ml polybrene. After two days infection, MSCs were selected in the presence of 0.4 mg/ml hygromycin B (Invitrogen) for 14 days, colonies of selected MSCs emerged. Then the pool stable cell lines designed as immortalized mesenchymal stem cells (IMSCs) were expanded and frozen in liquid nitrogen tanks. The reversal of IMSCs was achieved by adenovirus containing Cre (Ad-Cre) mediated site-specific Cre/loxP recombination. After infection with Ad-Cre for 48 h, western blotting was carried out to confirm SV40T was excised.

### Cell growth curve of primary mesenchymal stem cells and immortalized mesenchymal stem cells

Primary MSCs at passage 3 and IMSCs at passage 40 (7.5 × 10^4 ^per well) were inoculated into 6-well culture plates and incubated with complete DMEM/F12. Cells were trypsinized and counted with Trypan Blue exclusion assay (Sigma-Aldrich) as described [20] at 1, 2, 3, 4, 5 d after inoculation, respectively. Briefly, cell suspension was mixed with 2 × Trypan Blue solution. And then 10 μl of suspension mixture was added into hemocytometer and viable cells exclude trypan blue were counted twice under an inverted microscope (TE2000-S; Nikon). Three different independent experiments were executed in duplicate.

### Osteogenic differentiation, adipogenic differentiation and chondrogenic differentiation of primary mesenchymal stem cells and immortalized mesenchymal stem cells

Cells were seeded and grown to approximately 70-80% confluence in 6-well culture plates. To initiate trilineage differentiation, complete DMEM⁄ F12 medium was removed, and tissue-specific differentiation medium was added and maintained for three weeks as described [21-23]. Concretely, osteogenic induction medium (Hyclone) was added to induce osteogenic differentiation and subsequently changed every 3-4 days. Three weeks later, cells were fixed in methanol at -20°C for 20 min and stained with 1% Alizarin Red S (Sigma-Aldrich) for 20 min to measure calcified extracellular matrix. For adipogenic differentiation, cells were induced by adipogenic induction medium (Hyclone) which changed every 3-4 days. Three weeks later, cells were fixed and stained with 0.3% Oil Red O (Sigma-Aldrich) in isopropanol for 60 min. Chondrogenic induction medium was used to induce chondrogenic differentiation and changed every 3-4 days in the whole process. After 28 days, cells were fixed and stained with 1% Alcian Blue 8-GX (Sigma-Aldrich) in 0.1 M HCl (pH 1.0) at 25°C for 12 h to detect sulfated glycosaminoglycans deposited in matrix. Qualitative analysis was carried out under an inverted microscope.

### Neuronal induction of primary mesenchymal stem cells and immortalized mesenchymal stem cells

Firstly, cells were cultured in complete DMEM/F12 containing 1 μM all-*trans*-retinoic acid (ATRA) for 24 hr. When cultured cells confluenced approximately 70-80%, complete DMEM⁄ F12 was replaced by modified neuronal induction medium (MNM) [DMEM/F12/1.6% DMSO/160 μM butylated hydroxyanisole/20 mM KCl/1.6 mM valproic acid/8 μM forskolin/0.8 μM hydrocortisone/4 μg/ml insulin (all from Sigma)] to induce neuronal differentiation for 24 hr.

### Real-time PCR

Total RNA was extracted from cells with RNA isolation Kit (Genemega Inc.) according to the manufacturer's manual. The RNA sample was treated with DNase I (Invitrogen) to absolutely remove residual DNA. The first strand cDNA was generated from purified RNA sample using PrimeScript RT reagent Kit (TaKaRa) according to the manufacturer's instruction. Briefly, 2 μl 5 × PrimeScript Buffer, 0.5 μl PrimeScript RT Enzyme Mix I, 0.5 μl Oligo dT Primer, 0.5 μl Random 6 mers, and 500 ng total mRNA were mixed together to a total volume of 10 μl with RNA free water. The reverse transcriptase reaction was performed as follows: 37°C for 15 min then 85°C for 5 sec. The cDNA products were diluted 10-fold as PCR templates. Real-time PCR reaction was performed using RealMasterMix kit (SYBR Green; TIANGEN BIOTECH) and Bio-Rad Real-time PCR instrument. Reaction protocol was carried out as follows: 94°C × 20 sec, 55°C × 20 sec, 72°C × 20 sec, reading plate for 40 cycles. Data were represented as Ct value and calculated to the fold change using the ratio of the relative quantity of target gene to the β-actin. Primer sequences for Nestin, neuron specific enolase (NSE), microtubule-associated protein-2 (MAP-2), glial cell line-derived neurotrophic factor (GDNF) and β-actin were designed by using the Primer Primier 5 software and listed as follows:

Nestin, forward: 5'-GGGCAAGTGGAACGTAGA-3'

reverse: 5'-TCCCACCGCTGTTGATTT-3'

NSE, forward: 5'-CTGTTTGCTGCTCAAGGTC-3'

reverse: 5'-TCCCACTACGAGGTCTGC-3'

MAP-2, forward: 5'-GTATCAGGAGACAGGGAGGAG-3'

reverse: 5'-GGGGTAGTAGGTGTTGAGGTG-3'

GDNF, forward: 5'-CACTGACTTGGGTTTGGG-3'

reverse: 5'-TCACTTGTTAGCCTTCTACTTC-3'

β-actin, forward: 5'-TTTGAGACCTTCAACACCCC-3'

reverse: 5'-GGATGGCATGAGGGAGC-3'

### Immunofluorescence staining

Immunofluorescence staining was performed as described before [24]. Briefly, cells were fixed with methanol at -20°C for 20 min and washed twice with PBS. After blocked 1 hr with 5% BSA, the fixed cells were incubated with Nestin, neuron specific enolase (NSE), microtubule-associated protein-2 (MAP-2) (all from Abcam) and glial cell line-derived neurotrophic factor (GDNF) (Santa Cruz Biotechnology) primary antibody at 4°C overnight. After washed twice with PBS, all samples were incubated with appropriate secondary antibody conjugated to dylight 488 or 594 (Jackson ImmunoResearch Laboratories) for 1 hr. And then, 4'6'-diamidino-2-phenylindole dihydrochloride (DAPI) (Sigma) was used to stain cell nucleus. At last, all samples were examined and imaged under a fluorescence microscope (TE2000-S; Nikon). Control IgG of corresponding primary antibodies were used as negative controls.

### Western Blotting

Western blotting was performed as described previously [25]. Briefly, total protein was extracted by specific extraction kit (Bioteke Co. Ltd.). Protein concentrations of different samples were determined by using the BCA protein assay kit (Bioteke Co. Ltd.) and enzyme-labelling measuring instrument (Thermo Fisher Scientific). All protein samples were denatured by boiling and loaded onto a 10% SDS-polyacrylamide gel (Beyotime) with approximately 30 μg total proteins per lane. After electrophoretic separation, proteins were transferred to a polyvinylidene fluoride membrane (Millipore). After rinsed twice by tris buffered saline with tween-20 (TBST) (5 min per time), the membrane was blocked with 5% skimmed milk in TBST at 37°C for 1 h and probed with anti-NSE (Abcam) and anti-β-actin (Santa Cruz Biotechnology) primary antibodies at 4°C overnight then incubated with respective secondary antibody conjugated with horseradish peroxidase (Santa Cruz Biotechnology) at room temperature for 1 hr. The membrane was developed with an enhanced chemiluminescent kit (TIANGEN) and photographed by ECL Imaging System (Syngene GBOX, UK).

### Electrophysiological recordings

Resting membrane potential of cells was recorded using the whole-cell patch clamp technique at room temperature according to the methods described previously [26]. Cells were seeded on 8 × 8-mm glass coverslips and then cultured in complete DMEM⁄ F12 or induced by MNM for 24 h before electrophysiology recording. Coverslips were placed in an acrylic chamber (RC-26L) of inverted microscope (TE-2000U, Nikon, Japan), then perfused at 1.0-1.8 ml/min. Borosilicate glass capillaries (TW150F-4, WPI, FL, USA) were calcined and stretched into approximately 1 μm tip diameter patch electrodes. Pipette resistances approximately ranged from 3 to 4 m Ω. Electrodes were filled with internal solution containing (in mmol/L) 140 KF, 10 4-(2-hydroxyethyl)-1-piperazineethanesulfonic acid (HEPES), and 10 ethylene glycol tetraacetic acid (EGTA) and 5 adenosine triphosphate (ATP)-Na2. Internal solutions were adjusted to pH 7.2 with CsOH. The external bath solution contained (in mM) 140 NaCl, 5 KCl, 1.8 CaCl2, 1 MgCl2·6H2O, 10 D-glucose, and 10 HEPES were regulated to pH 7.4 with NaOH. After the formation of a gigaohm seal by negative pressure suction on single cell using patch electrodes, the whole-cell recording was established. Data acquisition was achieved by using Multiclamp 700B amplifier (Axon Instruments) and Digidata 1322 interface (Axon Instruments).

### Animals

Animal experiment protocols were approved by Chongqing Medical University Institutional Animal Care and Use committee. Sprague Dawley (SD) rats and nude mice were gained from the Experimental Animal Centre of Chongqing Medical University [certificate: SCXK (Yu) 2007~0001]. All rats and mice were housed under specified pathogens free (SPF) level laboratory conditions. Rats and mice were maintained under optimal conditions of hygiene, temperature, humidity, photoperiods (12L: 12D) with food and water available *ad libitum*.

### Tumorigenicity detection of primary mesenchymal stem cells and immortalized mesenchymal stem cells

Nude mice were used to detect tumorigenicity of primary MSCs and IMSCs. Cells suspended in PBS were transplanted into nude mice by subcutaneous injection. Rat yolk sac tumor cells (L2RYC) were used as positive control. Twenty four nude mice aging 8 weeks were divided into three groups equally. One group nude mice were subcutaneously injected primary MSCs (injected in the left front and rear notum of nude mice) and IMSCs (injected in the right front and rear notum of nude mice). Second group nude mice were subcutaneously injected rat yolk sac tumor cells (injected in the left front and rear notum of nude mice) and primary MSCs (injected in the right front and rear notum of nude mice). Third group nude mice were subcutaneously injected rat yolk sac tumor cells (injected in the left front and rear notum of nude mice) and IMSCs (injected in the right front and rear notum of nude mice). 1 × 10^6 ^cells were transplanted at each injection site. Six weeks later, all nude mice were executed by CO_2 _asphyxiation. Pictures of nude mice and gross specimen were taken using digital camera. Tumor nodules were removed and fixed in 4% paraformaldehyde solution. After fixation and paraffin embedded, tissues were cut into 5 μm thick sections. These tissue sections were deparaffinized in xylene and rehydrated using graded ethanol concentrations. The slides were stained by hematoxylin and eosin successively. And then, they were dehydrated and differentiated by ethanol. After dropping Canada balsam on the sections, coverslips were covered to seal them. Histological images were photographed with a microscope (Nikon ECLIPSE 55i, Japan) and a digital camera (LEIKA DFC 420C, Germany).

### Hypoxic-ischemic brain-damage (HIBD) rat model

Neonatal hypoxic-ischemic brain damage model was established as previous methods [27]. Briefly, the left carotid artery of seven day postnatal SD rat was isolated and ligated doubly. After 2 hours recovery with their mothers, offsprings were placed into a transparent plastic container which was ventilated with a constant flow of humidified mixture of 8% oxygen and 92% nitrogen for 2.5 hr. The container was placed in a 37°C water bath for maintaining proper temperature. 24 hr after hypoxia-ischemia, the offsprings were randomly divided into 3 groups and transfused with 1-2 × 10^6 ^MSCs or IMSCs in 100 μL of PBS by intraperitoneal injection.

### Morris Water Maze test

At age of five weeks, 15 rats of each group were received Morris water maze [28] test training to evaluate their spatial learning and memory abilities. Morris water maze test system (MWM SLY-WMS 2.0, China) was used in the test. The maze was a 1.5-m-diam circular pool filled with clear tap water at a temperature of 20 ± 0.5°C. A 10-cm-diam platform was placed in the III quadrant 1 cm up or submerged 2 cm below the water surface during visible test and invisible test. The procedure consisted of 1 d of visible platform tests and followed by 4 d of hidden platform tests, plus a probe trial 24 h after the last hidden platform test. In the visible platform test and the hidden platform tests, rats were tested for 4 contiguous trials in respective four quadrants per day; with an intertrial interval of 30 min. Track of animal movement was recorded in the test. To assess memory consolidation, a probe trial was performed after the last hidden platform test. In this trial, the platform was removed from the pool and the pass times in target area were recorded within 60 s.

### Shuttle Box test

At 35 days of age, 15 rats of each group were subjected to a shuttle box test (KE ZH-DSX2, China), comprising two compartments (50 × 20 × 25 cm). Experiments containing training and formal testing were performed to train the rats that consistent response to the electric shock. For each trial, a 10 sec conditioning stimulus (CS), comprising a buzzer and a light, was followed by 10 sec of unconditioned stimulus, comprising the CS plus a 0.6 mA electric current to the grid floor. All rats were trained for 1 day, 20 times a day, at intervals of 2-3 min. Formal test were operated at 2-5 d, 20 conditioning trials, separated by 2-3 min inter-trial intervals. If the rat run into the safe chamber within 10 s after sound and light, it was recorded for the active avoidance response, or fled into the safe chamber after got electric shocks, it was recorded for passive avoidance response; still not, it was record for no avoidance response. We analyzed the proportion of active avoidance response and no avoidance response, respectively.

### Statistical analysis

Results were expressed as Means ± S.E.M. if not indicated otherwise. The data were analyzed using SPSS 18.0 software. Statistical analyses were performed using one-way analysis of variance (ANOVA) or student *t *test. *P *≤ 0.05 was considered statistically significant.

## Results

### Isolation and identification of rat primary mesenchymal stem cells

**Figure 1:**
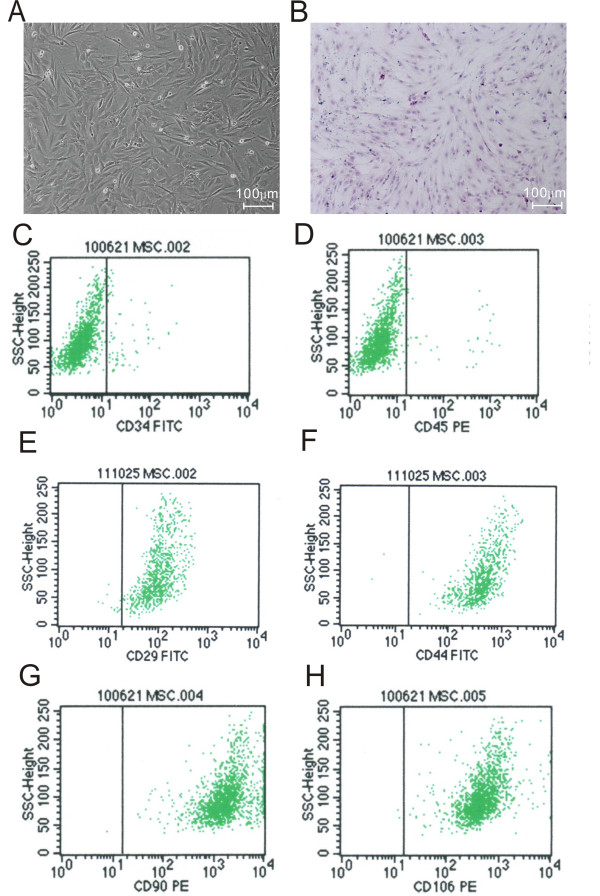
**Morphology and identification of rat primary mesenchymal stem cells (MSCs)**. A: Morphology of rat primary MSCs at passage 3 (Scale Bar = 100 μm). B: Cultured primary MSCs were stained with haematoxylin-eosin (HE) (Scale Bar = 100 μm). C-H: Primary MSCs (passage five) were analysed by FACS to detect the expression of mesenchymal surface markers using monoclonal antibodies. (C) CD34-fluorescein isothiocyanate (FITC) labeled; (D) CD45-Phycoerythrin (PE) labeled; (E) CD29-FITC labeled; (F) CD44-FITC labeled; (G) CD90-PE labeled; (H) CD106-PE labeled.

Primary MSCs were isolated from 4 weeks old SD rats and propagated *in vitro *successfully. MSCs adhered to the surface of plastic culture dishes and exhibited a spindle-shaped fibroblast-like morphology as cells approached confluence (Figure 1A, B). Although there is no specific marker, it is generally agreed that rat MSCs are positive for CD29, CD44, CD90 and CD106 and negative for CD34 and CD45. MSCs at passage 5 were identified by flow cytometry to detect these cell makers. As expected, more than 99% of the cells were CD29, CD44, CD90 and CD106 positive (Figure 1E, F, G, H), and vast majority of the cells were CD34 and CD45 negative (Figure 1C, D). These results were consistent with previous studies [29,30], confirming that these cells used in the present study were indeed MSCs without hematopoietic stem cells contamination.

### Construction and identification of immortalized mesenchymal stem cells

**Figure 2:**
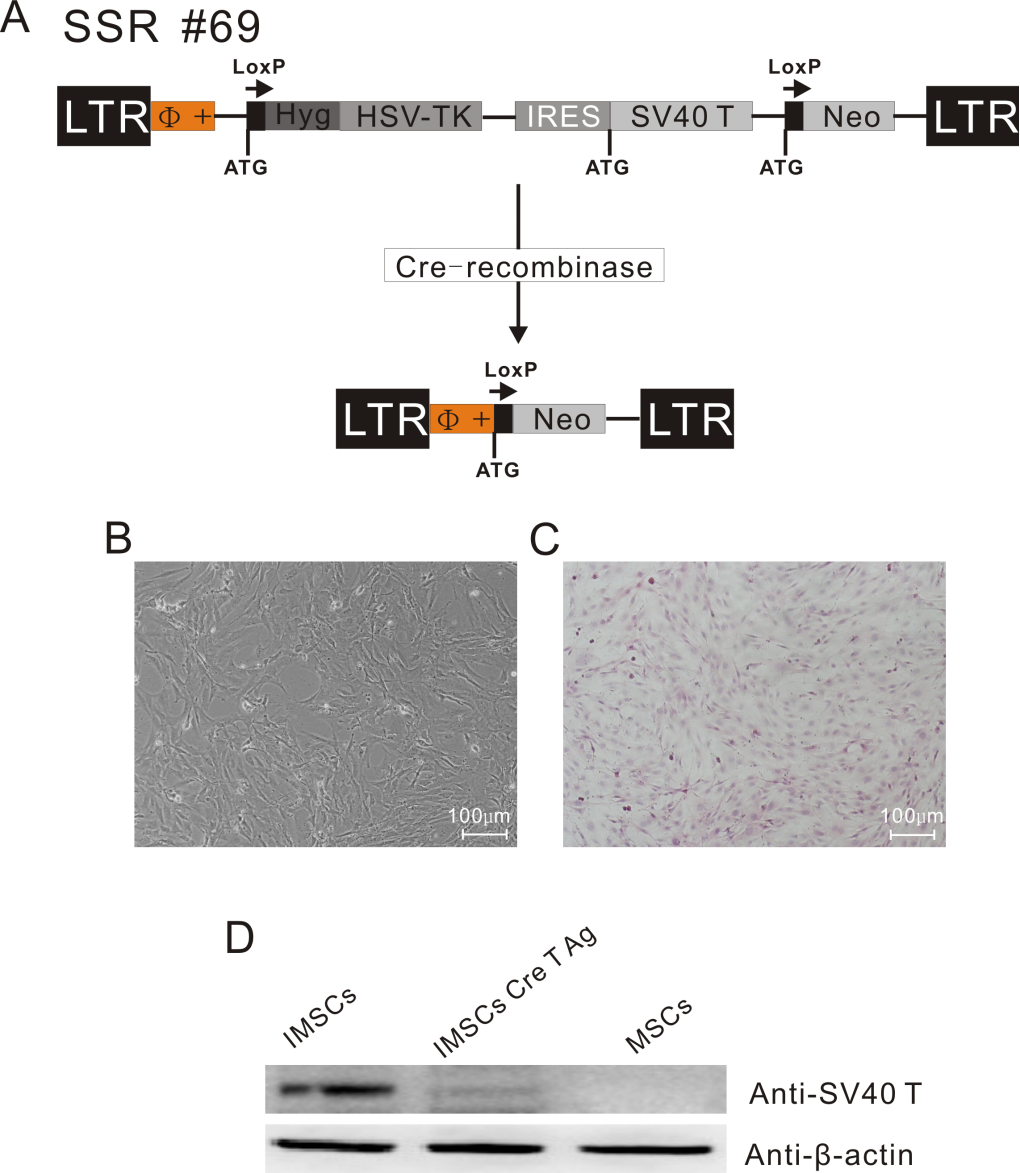
**Construction, morphology and characteristics of rat immortalized mesenchymal stem cells (IMSCs)**. A: Schematic drawings of the integrating component of retroviral vector SSR#69 for reversible immortalization. SSR#69 contains the hygromycin B resistance gene (Hyg R) as a positive selectable marker and the herpes simplex virus thymidine kinase gene (HSV-TK) as a negative selectable marker. The SV40T, Hyg R and HSV-TK genes are flanked by loxP sites. B: Morphology of rat IMSCs at 40 passage (Scale Bar = 100 μm). C: Cultured IMSCs were stained with haematoxylin-eosin (HE) (Scale Bar = 100 μm). D: Western bloting was performed to determine the protein level of SV40T antigen in three different kinds of MSCs. SV40T antigen expressed highly in IMSCs, however, it almost could not be detected in IMSCs after Cre-recombination. MSCs did not express SV40T antigen.

**Figure 3:**
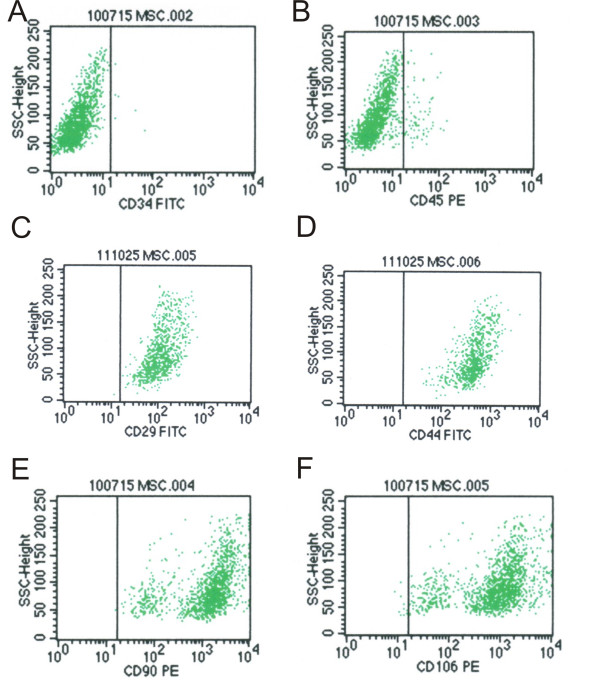
**Identification of rat immortalized mesenchymal stem cells (IMSCs) by FACS**. IMSCs were analysed by FACS to detect the expression of mesenchymal surface markers using monoclonal antibodies. (A) CD34-fluorescein isothiocyanate (FITC) labeled; (B) CD45-Phycoerythrin (PE) labeled; (C) CD29-FITC labeled; (D) CD44-FITC labeled; (E) CD90-PE labeled; (F) CD106-PE labeled.

The retroviral vector SSR#69 that expresses SV40T antigen and hygromycin resistant gene flanked with loxP sites (Figure 2A) was used to construct IMSCs. The retroviral vector SSR#69 can be reconstructed by site-specific Cre/loxP recombination to excise SV40T gene (Figure 2A). Primary MSCs were infected with retrovirus packaged SSR#69 for 3 days and selected with hygromycin B for 14 days. And then, MSCs which expresses SV40T and hygromycin resistant gene were obtained. These IMSCs at passage 40 were not different from primary MSCs in morphology and also presented spindle-shaped fibroblastic shape (Figure 2B, C). Western bloting result revealed that SV40T was highly expressed in IMSCs, but lost its expression after Cre/loxP mediated-excision, indicating that immortalization can be reversed (Figure 2D). Flow cytometry results showed IMSCs were positive for CD29 (99.81%), CD44 (99.96%), CD90 (99.84%) and CD106 (99.45%), and negative for CD34 (0.41%) and CD45 (4.27%) (Figure 3), indicating that MSCs surface makers did not change after immortalization. These data demonstrated that reversible IMSCs were constructed successfully and had the similar morphology and cell surface makers as primary MSCs.

### Proliferation capacity and trilineage potential of immortalized mesenchymal stem cells

**Figure 4:**
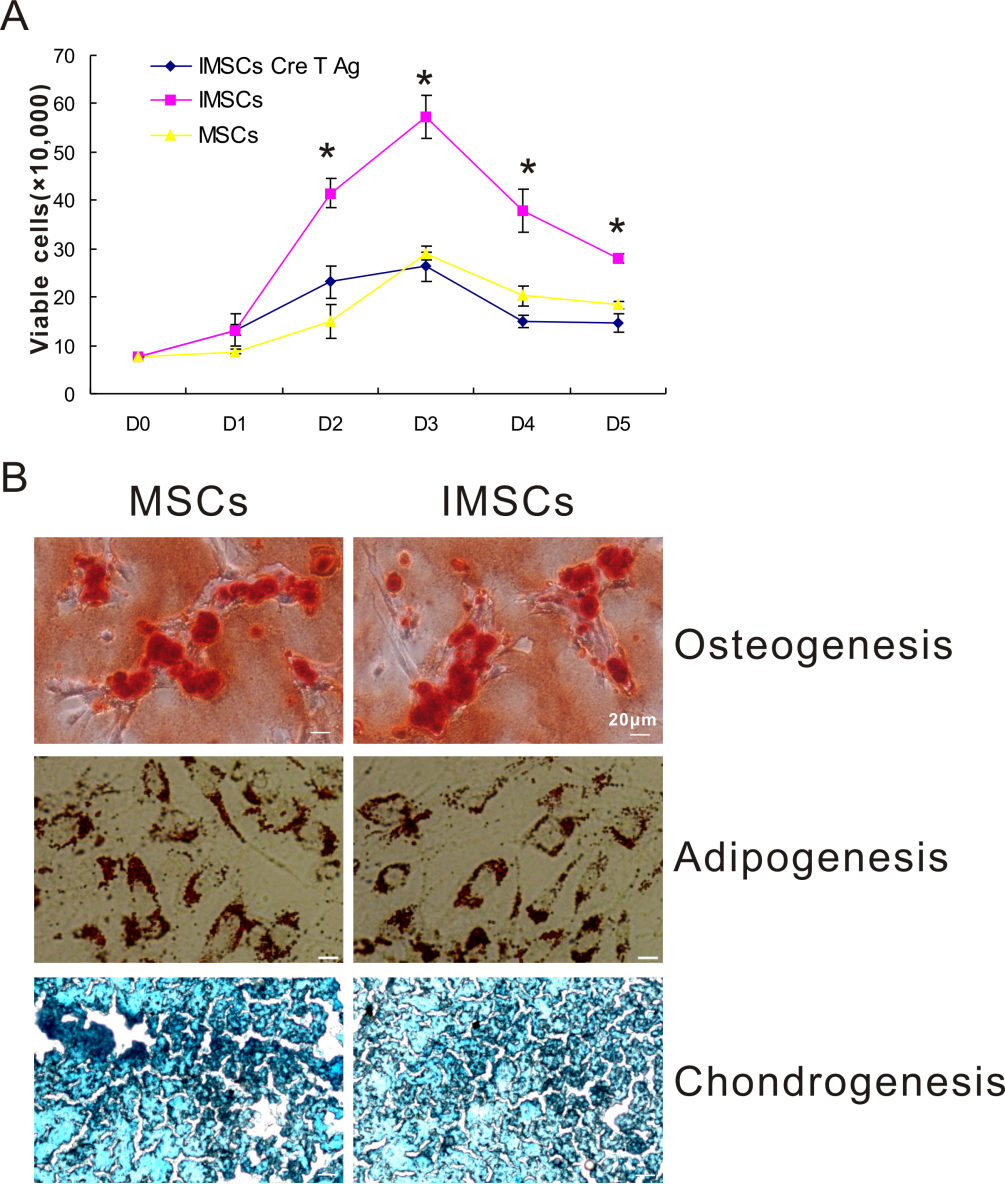
**Proliferation capacity of three different kinds of MSCs and trilineage potential of IMSCs**. A: Growth curve of three different kinds of MSCs (primary MSCs, IMSCs, and IMSCs Cre T antigen). The number of viable cells gradually increased from D0 and attained peak at D3 in all three kinds of MSCs. And then, viable cells number decreased sharply from D3 to D5. The viable cells number of IMSCs was significantly more than another two kinds of MSCs from D2 to D5 (*, *p *< 0.01 by one-way ANOVA). The tendence of cell growth of IMSCs was reversible with cre recombination. B: Osteocytes, chondrocytes and adipocytes were induced from primary MSCs and IMSCs. IMSCs displayed the same mesodermal trilineage differentiation potential as MSCs (Scale Bar = 20 μm).

To investigate whether immortalization by SV40T affects proliferation and differentiation potential of MSCs, cell growth curve determination and mesodermal trilineage differentiation tests were performed. The numbers of viable cells were counted at different time-points. All cells number continuously increased as time gradually went by, and arrived at peak at day 3. But it decreased to a platform from day 3 to day 5 (Figure 4A). As cell growth curve presenting, the number of viable IMSCs was significantly higher than that of primary MSCs from day 2 to 5 (*p *< 0.01). But the number of reversed IMSCs was not statistically different from that of primary MSCs from day 2 to 5 (*p *> 0.05). These data indicated that proliferation capacity of IMSCs was stronger than that of primary MSCs and could be reversed.

We then further determined whether IMSCs are able to maintain their mesodermal trilineage differentiation potential after immortalized by SV40T. As shown in Figure 4B, both MSCs and IMSCs could be induced *in vitro *to possess the typical characteristics of mature osteocytes, adipocytes, and chondrocytes, indicating that IMSCs maintain mesodermal trilineage potential even through immortalized by SV40T.

### Neuronal differentiation characteristics of immortalized mesenchymal stem cells *in vitro*

**Figure 5:**
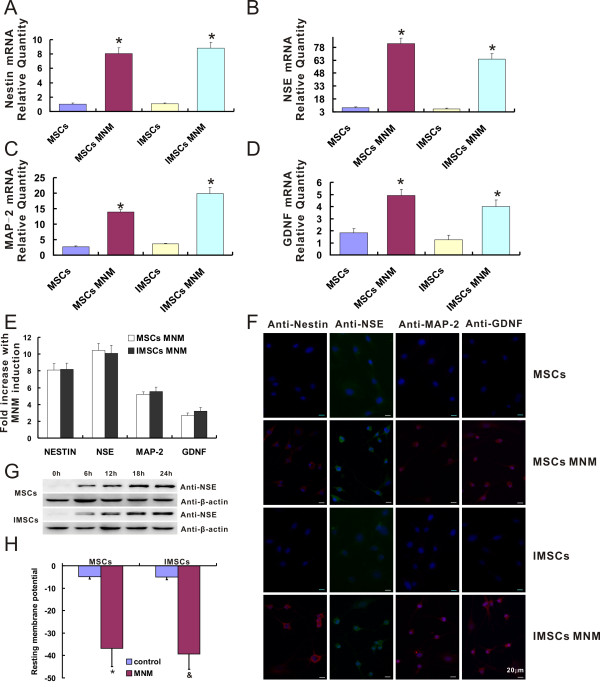
**IMSCs exhibit the same neuronal differentiation capacity as primary MSCs *in vitro***. A-D: Transcriptional expression of Nestin, NSE, MAP-2, GDNF during neuronal differentiation of MSCs and IMSCs. The transcriptional expression of genes was determined by measuring their mRNA levels with real-time PCR. Treatment of primary MSCs and IMSCs with MNM resulted in a dramatic increase in the mRNA levels (*, *P *< 0.001 by Student's *t *test). Real-time PCR results were confirmed in at least three batches of independent experiments with β-actin normalization. E: Fold increase of Nestin, NSE, MAP-2, and GDNF mRNA expression levels after induced by MNM. The mRNA expression levels of neural markers in both MSCs and IMSCs increased about 8, 10, 5 and 3 fold increases after treated with MNM, respectively (*P *> 0.05 by Student's *t *test). F: Immunofluorescence staining of Nestin, NSE, MAP-2, and GDNF during neuronal differentiation of MSCs and IMSCs (Scale bar = 20 μm). G: Expression of NSE during a 24 hr span including 0, 6, 12, 18, and 24 hr time-point of MNM induction in both primary MSCs and IMSCs were analyzed by western blotting. Equal loading of the samples was confirmed by comparable levels of β-actin detected in each lane. H: Resting membrane potential was measured by using whole cell patch clamp. The values represented Means ± S.E.M. (*, *p *< 0.001, MSCs vs. MSCs + MNM; ^&^, *p *< 0.001, IMSCs vs. IMSCs + MNM; *p *> 0.05, MSCs + MNM vs. IMSCs + MNM, by Student's *t *test).

Numerous studies have demonstrated that MSCs could be induced to differentiate into neuronal cells *in vitro *[31]. To investigate whether immortalization by SV40T affects neuronal differentiation of MSCs, comparison experiments about neuronal differentiation of primary MSCs and IMSCs were performed *in vitro*. As shown in Figure 5A-D, the mRNA levels of neural-specific genes including Nestin, NSE, MAP-2, and GDNF were significantly increased by 2-10 times after induced by MNM both in primary MSCs and IMSCs (*P *< 0.001). And the fold increases of these neural-specific genes were not significantly different (*P *> 0.05) between MNM-induced primary MSCs and IMSCs (Figure 5E). These data indicated that both primary MSCs and IMSCs could be induced to neuronal cells *in vitro *and there was no difference in mRNA level of neural specific markers after neuronal differentiation.

Next, immunofluorescence staining and western blotting were also performed to detect the protein expression level of neural-specific makers. After induced by MNM, both primary MSCs and IMSCs were changed into neural-like cells presenting distinct neuronal morphologies including simple bipolar, large and extensively branched multipolar appearance (Figure 5F). Nestin, NSE, MAP-2, and GDNF could hardly be detected in both primary MSCs and IMSCs, but these neural-specific makers expressed intensely in neural-like cells derived from both primary MSCs and IMSCs *in vitro *(Figure 5F). And then, the protein expression level of NSE during a 24 h span including 0, 6, 12, 18, and 24 h time-point of MNM induction in both primary MSCs and IMSCs were analyzed by western blotting. NSE expression increased dramatically at 6 h post MNM induction and continued to increase for up to 24 h. And the growth trend was probably consistent in both primary MSCs and IMSCs after induced with MNM (Figure 5G).

Resting membrane potential (RMP), which refers to the constant potential difference between inside and outside of the unstimulated cell membrane, is the premise and foundation of neurons excitability. RMP in different types of cells was measured by using whole cell patch clamp as a functional index. As shown in Figure 5H, both primary MSCs and IMSCs had low RMP (n = 8, -4.825 ± 1.12091 mV and -5.05714 ± 1.11484 mV; *P *> 0.05). However, neural-like cells derived from both primary MSCs and IMSCs were significantly hyperpolarized with a RMP of -36.875 ± 8.13173 (n = 16, *P *< 0.001, MSCs + MNM vs. control MSCs) mV and -39.2857 ± 6.84871 mV (n = 16, *P *< 0.001, IMSCs + MNM vs. control IMSCs), respectively. But there was no difference in RMP between two groups. These data indicated that IMSCs had the same potential to develop into mature functional mature neurons as primary MSCs.

### Transplantation of immortalized mesenchymal stem cells replaces MSCs to improves memory deficits in rat model of neonatal hypoxic-ischemic brain damage

**Figure 6:**
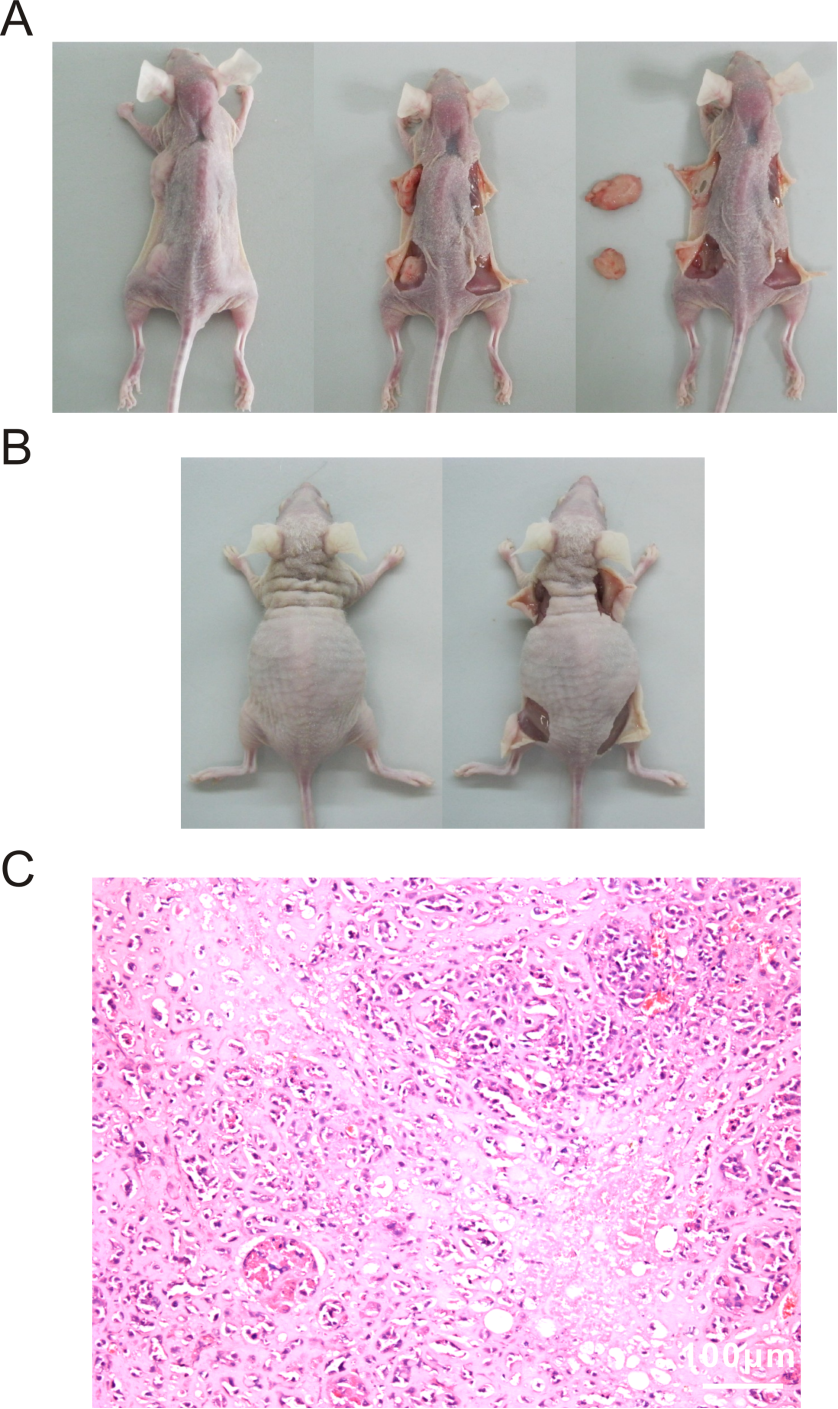
**Tumorigenicity detection of IMSCs**. A: Inoculated rat yolk sac tumor cell lines (L2RYC) performed peanut-sized bumps in injection sites (the left front and rear notum of nude mice), but no bump generated in IMSCs injection sites (the right front and rear notum of nude mice). B: No bump generated in both primary MSCs injection sites (the left front and rear notum of nude mice) and IMSCs injection sites (the right front and rear notum of nude mice). C: Haematoxylin-eosin (HE) staining for tumor nodules derived from L2RYC injection sites (Scale bar = 100 μm).

Transplantation experiments have demonstrated that MSCs therapy could improve neurological function in animal models of Parkinson's disease [32], stroke [33], cerebral ischemia [34], and spinal cord injury [35]. The functional recovery of the HIBD models after treatment of primary MSCs and IMSCs transplantation was compared by using Morris water maze and shuttle box tests. Before IMSCs transplantation, we excluded its tumorigenicity firstly. Primary MSCs, IMSCs and rat yolk sac tumor cell lines (L2RYC) were subcutaneously inoculated in nude mice, respectively. Six weeks later, tumorigeneses of these cells were investigated. As shown in Figure 6A and 6B, inoculated L2RYC performed peanut-sized bumps in injection sites; however, both MSCs and IMSCs had no bump formation in nude mice. And then positive bumps were removed and stained by hematoxylin-eosin. Many naïve morphology and atypia appearance of microcystic structure, Schiller-Duvall body, papillary and glomeruloid-like structures were observed in the bump tissues (Figure 6C). These data indicated that IMSCs were safe for transplantation therapy *in vivo *as primary MSCs.

**Figure 7:**
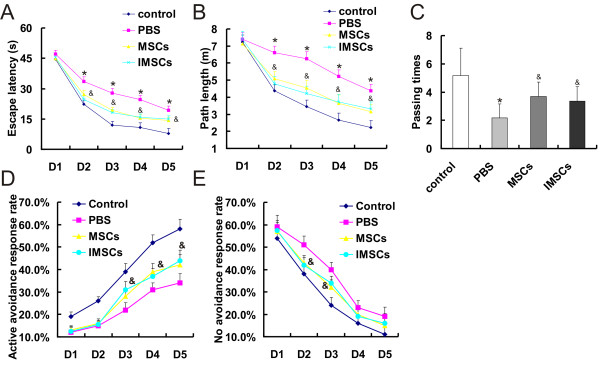
**Therapeutic effects of primary MSCs and IMSCs in rat model of neonatal HIBD by cell transplantation**. A: In hidden platform tests (from D2 to D5), the escape latency of PBS group (*, *p *< 0.01, PBS vs. control) became shorter after primary MSCs or IMSCs therapy (^&^, *p *< 0.05, MSCs/IMSCs vs. PBS). There were no statistical difference between primary MSCs group and IMSCs group (*p *> 0.05). B: In hidden platform tests (from D2 to D5), the path lengths of PBS group (*, *p *< 0.01, PBS vs. control) became shorter after primary MSCs or IMSCs therapy (^&^, *p *< 0.05, MSCs/IMSCs vs. PBS). There were no statistical difference between primary MSCs group and IMSCs group (*p *> 0.05). C: In the probe trial on the sixth day, the passing times of PBS group (*, *P *< 0.01, PBS vs. control) significantly increased after primary MSCs or IMSCs therapy (^&^, *P *< 0.05, MSCs/IMSCs vs. PBS). And the MSCs group and IMSCs group had similar passing times (*P *> 0.05). D: The active avoidance response rate (AARR) of the MSCs group and IMSCs group were significantly higher than that of PBS group on the 3rd, 4th and 5th test day (^&^, P < 0.05, MSCs/IMSCs vs. PBS). E: The no avoidance response rate (NARR) of MSCs group and IMSCs group were significantly lower than that of PBS group on the 2^nd ^and 3^rd ^test day (^&^, P < 0.05, MSCs/IMSCs vs. PBS).

The Morris water maze tests were performed four weeks after HIBD models received cells transplantation therapy. In the visible platform tests (D1), each group exhibited similar escape latencies (*P *> 0.05 by Student's *t *test; Figure 7A) and path lengths (*P *> 0.05 by Student's *t *test; Figure 7B), which indicated that HIBD treatment did not affect rat motility or vision. In the hidden platform-swimming test (from D2 to D5), the escape latencies and the path lengths of all groups gradually reduced during test time. PBS group showed significant decline compared with control group. The escape latency from D2 to D5 of PBS group was longer than that of control group rats (*, *p *< 0.01, PBS vs. control, by one-way ANOVA; Figure 7A). The PBS group rats had to swim significantly longer distances to reach the platform compared with control rats from D2 to D5 (*, *p *< 0.01, PBS vs. control, by one-way ANOVA; Figure 7B). However, the escape latency and path length became shorter after primary MSCs or IMSCs therapy (^&^, *p *< 0.05, MSCs/IMSCs vs. PBS, by one-way ANOVA; Figure 7A, B). Further more, the MSCs group and IMSCs group had similar escape latencies and paths length (*p *> 0.05 by one-way ANOVA; Figure 7A, B). In the probe trial on the last day of testing, the platform was removed. As shown in Figure 6F, compared with control group, there was significantly less number of the PBS group rats traveled through the place where the hidden platform was previously placed (*, *P *< 0.01, PBS vs. control, by Student's *t *test; Figure 7C). However, the passing times significantly increased after primary MSCs or IMSCs therapy (^&^, *P *< 0.05, MSCs/IMSCs vs. PBS, by Student's *t *test; Figure 7C). And the passing times between two cell therapy groups had no statistic difference (*P *> 0.05 by Student's *t *test; Figure 7C). Further, we examined learning ability through the shuttle box test. We found that HIBD rats received primary MSCs or IMSCs therapy showed an increased active avoidance response rate (AARR) on the 3^rd^, 4^th ^and 5^th ^test day (^&^, P < 0.05, MSCs/IMSCs vs. PBS, by one-way ANOVA; Figure 7D) and decreased no avoidance response rate (NARR) on the 2^nd ^and 3^rd ^test day (^&^, P < 0.05, MSCs/IMSCs vs. PBS, by one-way ANOVA; Figure 7E). In addition, both AARR and NARR between two cell therapy groups had no statistic difference (*p *> 0.05 by one-way ANOVA; Figure 7D, E). Taken together, these above data indicated that IMSCs were the same as primary MSCs to be used in repairing brain injury with improved learning ability and spatial memory.

## Disscussion

Aging has been defined as ''the sum of primary restrictions in regenerative mechanisms of multicellular organisms'' by Sames and Stolzing [36]. Cellular senescence is a complex phenotype that entails changes in both function and replicative capacity [37]. Because of its finite replicate capacity, MSCs will become senescent after multiple passages. The morphology and multilineage differentiation capacity of senescent MSCs are different from young MSCs. Since a tremendous number of cells are needed for a clinical application, MSCs must be expanded by consecutive passages to provide enough cell sources. A significant increase in the fraction of flattened/hypertrophic and multinucleated MSCs over consecutive passages [38,39] seriously affects its application value and therapeutic effects for cell-based regenerative medicine and tissue engineering. Simian virus 40 large T (SV40T) antigen which is derived from polyomavirus SV40 is capable of perturbing the retinoblastoma (pRB) and p53 tumor suppressor proteins and interfering with one or more specific cyclins [40]. It causes the cells to leave G1 phase and enter into S phase, which promotes DNA replication and cell proliferation. SV40T was used as a prototypical immortalizing gene [19].

Here, we show that an immortalized mesenchymal stem cells (IMSCs) line has been constructed by infected with retrovirus SSR#69 expressing SV40T. Consistent with previous reports, the morphology [41] and cell surface markers [42,43] of IMSCs did not change after consecutive 40 passages. Immortalization by SV40T prevented MSCs from senescence over consecutive passages, meanwhile maintained its surface marks. The retroviral vector SSR#69 is characterized by that can be reconstructed by site-specific Cre/loxP recombination to excise SV40T (Figure 2A). We revealed that immortalization could be reversed after Cre/loxP mediated-excision (Figure 2D). This characteristic of IMSCs ensures the safety of its clinical application. Several independent reports have shown that cells proliferation capacity can be enhanced by SV40T [44-46]. We found that proliferation capacity of IMSCs was significantly stronger than that of primary MSCs. The number of IMSCs can be expanded rapidly by limited passages in the short term to obtain gigantic number of cells and content the needs of experimental study both *in vitro *and *in vivo*. As previous studies reported that cells preserved their differentiation capacity after immortalization [47-51]; IMSCs in the present study also retain multilineage differentiation capacity including osteogenesis, adipogenesis, chondrogenesis and neurogenesis. In short, IMSCs not only retained their features of primary MSCs but also possessed higher proliferation capacity and anti-senescence ability.

The ability of MSCs to differentiate into functional neurons has been demonstrated by many *in vitro *studies [52], and further confirmed by cell transplantation experiments in brain injury and neurodegenerative diseases animal models [53,54]. Our previous studies have also supported those results [9,10]. Can IMSCs as an alternative to MSCs be used in neuronal differentiation research *in vitro *and transplantation experiments to improve neurological function of brain injury animal models? In the present study, we showed that IMSCs could definitely be induced to differentiate into neuronal cells *in vitro*. Meanwhile, neural-like cells derived from IMSCs had similar expressions of neural-specific genes, protein expression patterns and resting membrane potential (RMP) compared with their counterparts derived from primary MSCs. Consistent with primary MSCs, IMSCs could be induced into neuronal differentiation and had the same potential to develop into mature functional neurons *in vitro*. Although previous reports showed that immortalized hepatocytes prevented acute liver failure in animal models without tumorigenesis [55-57], SV40T mediated cells might be closely related to oncogenesis [58,59]. Before IMSCs transplantation in HIBD models, we firstly detected its tumorigenicity firstly. We found that there was no tumor formation in nude mice at 6-week after subcutaneously inoculated with IMSCs, indicating that IMSCs were safe for transplantation therapy *in vivo*. And then, we transplanted IMSCs into HIBD models to investigate its impact on neurological function. The Morris water maze (MWM) and shuttle box are frequently used laboratory tools for detecting learning ability and spatial memory of animal models [60,61]. In animal behavioral tests, we demonstrated that IMSCs possessed the same effect as primary MSCs in improving the cognitive function in HIBD rats. Before we transplanted MSCs into HIBD rats, we have verified that GFP labeled primary MSCs and IMSCs transplanted by intraperitoneal injection could migrate and locate in the brain region (data not shown). The neurological function recovery of HIBD rats might be closely related to the transdifferentiation or immuno-regulation effects of MSCs. Our studies suggested that IMSCs was indeed a great alternative for MSCs in neuronal differentiation research *in vitro *and transplantation experiments in brain injury animal models.

## Conclusion

In summary, we have established an immortalized mesenchymal stem cells line with the characteristic of reversible immortalization. IMSCs possessed higher proliferation capacity and anti-senescence ability in addition to fundamental features of primary MSCs. IMSCs were proved to be a potential and valuable alternative to MSCs in neuronal differentiation and neuroregeneration associated studies.

## Abbreviations

MSCs: mesenchymal stem cells; IMSCs: immortalized mesenchymal stem cells; SV40T: simian virus 40 large T; HIBD: hypoxic-ischemic brain damage; MNM: modified neuronal induction medium; NSE: neuron specific enolase; MAP-2: microtubule-associated protein-2; GDNF: glial cell line-derived neurotrophic factor; RMP: resting membrane potential.

## Competing interests

The authors declare that they have no competing interests.

## Authors' contributions

MG, YB, JC and TL designed research. MG and YB performed experiments. WJ, YZ, LC, NH and XW helped MG in experiments. MG, JC and YL analyzed the data. MG, YB, JC and TL wrote the paper. All authors read and approved the final manuscript.
